# Students learning performance prediction based on feature extraction algorithm and attention-based bidirectional gated recurrent unit network

**DOI:** 10.1371/journal.pone.0286156

**Published:** 2023-10-25

**Authors:** Chengxin Yin, Dezhao Tang, Fang Zhang, Qichao Tang, Yang Feng, Zhen He

**Affiliations:** 1 Institute of Vocational Education, Chengdu Aeronautic Polytechnic, Chengdu, Asia, China; 2 Faculty of Education, Beijing Normal University, Beijing, Asia, China; 3 College of Information Engineering, Sichuan Agricultural University, Yaan, Asia, China; Jeonbuk National University, KOREA, REPUBLIC OF

## Abstract

With the development of information technology construction in schools, predicting student grades has become a hot area of application in current educational research. Using data mining to analyze the influencing factors of students’ performance and predict their grades can help students identify their shortcomings, optimize teachers’ teaching methods and enable parents to guide their children’s progress. However, there are no models that can achieve satisfactory predictions for education-related public datasets, and most of these weakly correlated factors in the datasets can still adversely affect the predictive effect of the model. To solve this issue and provide effective policy recommendations for the modernization of education, this paper seeks to find the best grade prediction model based on data mining. Firstly, the study uses the Factor Analyze (FA) model to extract features from the original data and achieve dimension reduction. Then, the Bidirectional Gate Recurrent Unit (BiGRU) model and attention mechanism are utilized to predict grades. Lastly, Comparing the prediction results of ablation experiments and other single models, such as linear regression (LR), back propagation neural network (BP), random forest (RF), and Gate Recurrent Unit (GRU), the FA-BiGRU-attention model achieves the best prediction effect and performs equally well in different multi-step predictions. Previously, problems with students’ grades were only detected when they had already appeared. However, the methods presented in this paper enable the prediction of students’ learning in advance and the identification of factors affecting their grades. Therefore, this study has great potential to provide data support for the improvement of educational programs, transform the traditional education industry, and ensure the sustainable development of national talents.

## 1. Introduction

Equitable quality education is the key to promoting sustainable social development. UNESCO has made education an important goal of sustainable development in the 2030 Framework for Action on Education [1]. The education sector should provide an inclusive, equitable, and quality educational environment that promotes lifelong learning opportunities for all. Building a learning society necessarily gives priority to education. Truancy and dropout among students in secondary and tertiary education is a problem that deserves the attention of all. A high dropout rate results in a waste of teaching resources and can even lower the overall literacy level of society. In the context of sustainable social development, the wealth of educational data provides researchers with the opportunity to study students’ learning. The prediction of students’ performance is one of the most challenging areas. Among them, the analysis and prediction of learning achievements are important means to ensure teaching quality and an important method to ensure students’ future development.

The existing research shows that grade is the primary carrier of students’ performance. Predicting potential performance in advance through student-related personal attributes and learning behaviors can help build a quality environment that promotes academic progress. Models such as eXtreme Gradient Boosting (XGBoost) [2], Light Gradient Boosting Machine (LightGBM) [3], and GRU have a wide range of applications in forecasting. As one of the key factors in identifying students’ attributes, such as social status, school regulations and psychological qualities, external attributes can impact students’ performance [4]. In terms of the evaluation of external attributes, Natek et al. further took factors such as student gender and family background into account factors [5]. It has been found that background factors in external attributes can help assess whether students can succeed before they have a learning experience. Therefore, family background plays an important role in students’ attributes [6]. In addition, internal attributes, such as homework grades and regular tests are equally important. Students’ performance and learning behaviors have a direct impact on learning results such as usual tests, and monthly exams usually affect the final grades. If intelligent methods are applied to identify disadvantaged students, and help them improve their academic performance, it will prevent these students from dropping out of school and improve retention rates [7].

The prediction data used in the experiment were all obtained from the UCI Machine Learning Repository [8], including 395 students’ mathematics grades and 649 Portuguese language grades with 33 indicators. The prediction of students’ grades is based on students’ learning performance and multiple background characteristics. A total of 30 key questions are extracted from this information to form the questionnaire. The purpose of prediction is to estimate the unknown value of a variable based on important historical data and relevant data. Therefore, when predicting the final performance of students, it is also necessary to consider the students’ previous grades, including the grades of the first stage and the second stage. The data provided in this paper should meet the following research objectives:

O1: Convert the text information in the original data into digital information through dictionary mapping, so as to complete the preprocessing between data.
O2: Using the Pearson correlation coefficient method to analyze the correlation between data.
O3: Perform feature processing on the data through data mining algorithms, and select several groups of data with high impact weight on the target variable.
O4: Analyze the prediction effect of the model through various judgment indicators.

Through the analysis of relevant data, this paper answers the questions such as which factors have the greatest influence on learners, what learners need most, and whether more accurate prediction models can be found in the learning field. Based on the analysis, this paper provides data support for policy suggestions and educational projects. The innovations of this paper are as follows: (1) Using a time series prediction method to study students’ performance data, instead of simple classification prediction. (2) The prediction effect of the model is improved by considering the interaction between pre-grade and post-grade. (3) This paper compares the prediction effects of models based on various data mining methods and selects the factor analysis model as the final feature extraction method. (4) The FA-BiGRU-attention model has achieved the best prediction effect in this public data set when compared with other models.

The remainder of this paper is organized as follows: section 2 summarizes the literature review on the application of data mining and machine learning in education; section 3 describes the data sources, data analysis, the judgment of the predictive effect, and the final experimental methodology; section 4 details the various steps of the experiment and the predicted results; section 5 answers the questions mentioned in the literature review section, discusses the study limitations and future research directions.

## 2. Literature review

Data mining techniques can analyze more valuable information hidden in a large number of datasets [9]. Educational data mining is to know and analyze the situation of students from educational data through learning analysis tools [10]. Therefore, we investigated the application of various data mining techniques and machine learning models in the field of education. Kaur et al identified the groups with low learning efficiency among students by using a predictive data mining model based on classification algorithms [11]. They expounded on the importance of data mining algorithms based on prediction and classification in the field of education. Costa et al used educational data mining (EDM) techniques to predict student failure rates in introductory programming courses at an early enough stage [12]. Abidi et al identified in advance the student group who would be confused when doing algebra homework by seven machine learning methods such as random forest (RF) and gradient boosting tree (XGBoost) to help them learn knowledge and develop talent [10]. Jokhan et al developed an early warning system (EWS) to predict student performance in an IT course at a university in the South Pacific area through the correlation between online behavior and grades, with a prediction accuracy of 60.8% [13]. Also Poddar et al used the principal component analysis (PCA) method to determine the three main components of factors such as guarantor, cargo safety, and train punctuality in railway transportation, and conducted factor analysis (FA) on the main components and found out the most important factors based on a single factor load [14]. Kim et al adopted data mining techniques to predict their ICT literacy level [15]. From the prediction results of OneR, J48, bagging, random forest, multi-layer perceptron and minimum sequential optimization (SMO) algorithm, they found that when there are 47 attributes, the SMO algorithm achieves the highest early prediction accuracy, with an accuracy rate of about 69%.

The machine learning algorithm is a booming technology, which shows great potential in the field of education. On the one hand, it helps teachers to identify students who are at risk of performance in their learning and proactively provide educational interventions to create a better learning environment for students [10]. On the other hand, it helps educational institutions to predict possible risk events in advance, thus giving them enough time to find measures to mitigate the impact. In our current work, our focus is on predicting students’ performance by mining education-related data.

Through historical data and multiple background feature data, machine learning can obtain and utilize the patterns among the data to study students’ final grades. By learning and training from data to analyze decision behaviors, machine learning can achieve the purpose of predicting a value in the future. For example, machine learning techniques such as the attention mechanism, random forest (RF), and bidirectional gated recurrent units (BiGRU) have been widely used in various fields of prediction tasks [16–18]. Niu and Xu demonstrated in their study of the stock price index that introducing the attention mechanism into GRU, i.e., assigning different weights to the input elements in advance, can improve the accuracy of the prediction level [19]. Jung et al applied recurrent neural networks (RNNs), long short-term memory (LSTM), and gated recurrent unit (GRU) networks to multi-step advance prediction of electric loads [20]. RNN and GRU can understand the previous point and predict the current point very well, but they assign the same weights to all variables, resulting in limited predictive power. Hence, Jung et al. changed to use the GRU model based on the attention mechanism, which allowed important variables to obtain higher weights and significantly improved the accuracy of model predictions. Veeramsetty et al proposed a gated recurrent unit (GRU) machine learning model based on random forest (RF) to predict power load [21]. This model adopts RF to reduce the input dimensionality of the model to form a lightweight GRU model. Compared with the original GRU, the GRU model combined with RF will reduce the calculation time and storage space, and the author proved the effectiveness of the model in a random environment. Li et al used the GRU neural network to predict wave heights at six different stations along the Chinese coast in the short-term and long-term, respectively [22]. The experimental results showed that for the 1-hour advance forecast, GRU is better than other methods in predicting all indexes. For the 3-hour advance forecast, the GRU network shows stronger robustness than the LSTM network.

Therefore, the practical application of educational data mining and the machine learning algorithm has changed the traditional teaching model, optimized its effectiveness and improved its quality.

## 3. Methodology

**Fig 1:**
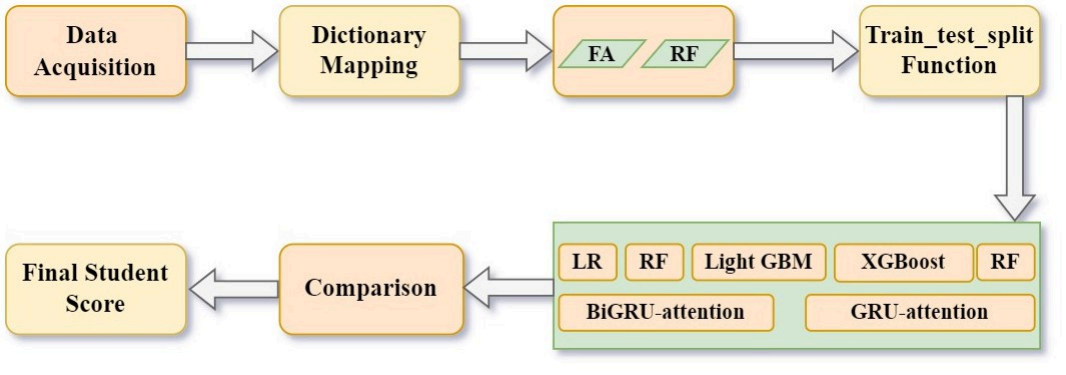
The framework of the methodology.

The methodology of this article includes the following steps: data acquisition, data processing, feature selection, applying machine learning and comparing forecast effect. The framework of the detailed methodology can be seen in Fig 1.

### 3.1. Data

#### 3.1.1. Data processing

The original data in the questionnaire contains both text information and digital information, so the final experimental results will be affected if the data are imported directly into the computer for training. To better predict student learning performance, the text information needs to be pre-processed before the experiment. The pre-processing text information is generally divided into one-hot encoding and dictionary mapping. Given that factors such as feature distance and feature size in one-hot encoding are not relevant to the model’s training, this article only needs to perform dictionary mapping on the information [23]. Table 1 shows the information and explanations of 33 characteristic attribute variables related to students’ learning performance. In the specific performance of students’ grades, 0 is the lowest and 20 is the most perfect.

**Table 1:** Characteristic attributes of data.

Order	Feature Name	Description	Correlation with G3 in Math	Correlation with G3 in Por
O1	School	Student’s school	-0.045	-0.2842
O2	Sex	Student’s gender	-0.1035	-0.1291
O3	Age	Student’s age	-0.1616	-0.1065
O4	Address	Type of student’s home address	-0.1058	-0.1676
O5	Famsize	Student’s family size	0.0814	0.045
O6	Pstatus	parent’s cohabitation status	-0.058	-0.0008
O7	Medu	Student’s mother’s education	0.2171	0.2401
O8	Fedu	Student’s father’s education	0.1525	0.2118
O9	Mjob	Student’s mother’s job	0.102	0.0166
O10	Fjob	Student’s father’s job	-0.0029	-0.0333
O11	Reason	Reasons for choosing this school	0.1205	0.0346
O12	Guardian	Student’s guardian	-0.0415	-0.0295
O13	Traveltime	Travel time from home to school	-0.1171	-0.1272
O14	Studytime	Student’s weekly study time	0.0978	0.2498
O15	Failures	Number of class failures in past	-0.3604	-0.3933
O16	Schoolsup	Additional educational support	-0.0828	-0.0664
O17	Famsup	Educational support from family	-0.0392	0.0592
O18	Paid	Extra paid classes within the course subject	0.102	-0.0549
O19	Activities	Extra-curricular activities	0.0161	0.0598
O20	Nursery	Attended nursery school	0.0516	0.0288
O21	Higher	Desire of taking a higher education	0.1825	0.3322
O22	Internet	Internet access at home	0.0985	0.15
O23	Romantic	Have a romantic relationship	-0.13	-0.0906
O24	Famrel	Quality of family relationships	0.0514	0.0634
O25	Freetime	Free time after school	0.0113	-0.1227
O26	Gout	Going out with friends	-0.1328	-0.0876
O27	Dalc	Alcohol usage at daytime	-0.0547	-0.2047
O28	Walc	Alcohol usage at weekend	-0.0519	-0.1766
O29	Health	Current health status	-0.0613	-0.0989
O30	Absences	Number of student’s absences	0.0342	-0.0914
O31	G1	First period grade	0.8015	0.8264
O32	G2	Second period grade	0.9049	0.9185
O33	G3	Final grade	1	1

Through the train_test_split function, the data is divided into a test set, and a training set in the ratio of 8:2 and then is put into the model to train and get the prediction results. Table 2 shows the digital conversion form of some mathematical grade data after dictionary mapping.

**Table 2:** Numeric conversion of textual data.

School	Sex	Age	Adless	Famsize	Pstatus	Medu	Fedu	⋯	G1	G2	G3
0	1	18	0	0	0	4	4	⋯	5	6	6
0	1	17	0	0	1	1	1	⋯	5	5	6
0	1	15	0	1	1	1	1	⋯	7	8	10
0	1	15	0	0	1	4	2	⋯	15	14	15

### 3.2. Feature selection

In addition to students’ final performance, the dataset contains 30 groups of characteristic data and two groups of performance data. In order to judge the relationship between each column of data and the final grade, the G3 data needs to be analyzed by the Pearson correlation coefficient method before the experiment. The analysis results of the correlation coefficient are shown in Table 1, and the calculation formula is as follows:

$$\begin{array}{c} \rho_{XY}=\frac{\sum_{i=1}^{n}(X_{i}-\bar{X})(Y_{j}-\bar{Y})}{\sqrt{\sum_{i=1}^{n}{\left(X_{i}-\bar{X}\right)}^{2}\sqrt{\sum_{j=1}^{n}{\left(Y_{j}-\bar{Y}\right)}^{2}}}} \end{array}$$ (1)

Where *X* = [*X*_1_, *X*_2_, ⋯, *X*_*i*_, ⋯, *X*_*n*_],*Y* = [*Y*_1_, *Y*_2_, ⋯, *Y*_*i*_, ⋯, *Y*_*n*_];$\bar{X}$ and $\bar{Y}$ are the mean values of samples in two feature sets respectively; *ρ*_*XY*_ is the degree of correlation between variables *X* and *Y*. The closer the absolute value of *ρ*_*XY*_ is to 1, the higher the degree of correlation between the two variables. It can be observed from Table 1 that except G1 and G2 have high degree of correlations with G3 and are both positive correlations, other characteristic attributes such as Pstatus have no obvious correlation with G3. Considering that too many feature attributes will affect the prediction effect of the model [24], this paper uses the RF algorithm to extract features from experimental data.

The random forest can process high-dimensional data and sort characteristic attribute variables according to their importance. [25] By calculating the measurement value of each decision tree, that is, the prediction error value of any characteristic attribute variable whose ranking order changes when observed outside the bag. The Variable Importance (VI) is obtained by dividing the average value of all tree-measured values by the standard deviation of all decision tree-measured values. The formula for Variable Importance is as follows:

$$\begin{array}{c} VI=\frac{\frac{1}{n}\sum_{i=1}^{n}(errOOB_{i}^{\prime }-errOOB_{i})}{\sqrt{\frac{1}{n}\sum_{1}^{n}{\left(errOOB_{i}-errOOB_{i}\right)}^{2}}} \end{array}$$ (2)

Where n is the number of trees; *errOOB*_*i*_ is the out-of-bag sample error for the ith tree; $errOOB_{i}^{\prime }$ is the out-of-bag sample error for the ith tree when the variable ordering is changed; *errOOB*_1_ is the average out-of-bag sample error. The higher the VI value, the higher the importance of the characteristic attribute variable.

### 3.3. Performance index

To study the prediction effect of students’ learning performance, this paper uses the value of the determined coefficient *R*^2^ to analyze the fitting of students’ real grades and predicted grades. The prediction errors of each model can also be calculated by using the root mean square error (RMSE) and the mean absolute error (MAE). The larger the *R*^2^ value is, the more the fitting between the real value and the predicted, and the smaller the error value is, the better the prediction method of the model will be. The calculation formulas of *R*^2^, RMSE and MAE are as follows:

$$\begin{array}{c} R^{2}=\frac{\sum {\left(\hat{y_{t}}-\bar{y_{t}}\right)}^{2}}{\sum {\left(y_{t}-\bar{y_{t}}\right)}^{2}} \end{array}$$ (3)

$$\begin{array}{c} RMSE=\sqrt{\frac{1}{N}\sum_{t=1}^{N}(y_{t}-\hat{y_{t}})} \end{array}$$ (4)

$$\begin{array}{c} MAE=\frac{1}{N}\sum_{t=1}^{N}|y_{t}-\hat{y_{t}}| \end{array}$$ (5)

Where *y*_*t*_ represents the real value; $\hat{y_{t}}$ represents the predicted value; $\bar{y_{t}}$ represents the average value of the real value, and *N* represents the total number of data.

### 3.4. FA-BiGRU-attention model

To predict students’ learning performance in mathematics and Portuguese courses accurately, this paper continues to do research on students’ grade data through the FA-BiGRU-attention model.

#### 3.4.1. Factor analysis model

The idea of the factor analysis model is to solve for correlations between indicators, so that data indicators with high correlations are classified as the same group and indicators with weak correlations as another group. The correlation coefficient matrix is used to find a few common factors to describe the relationship between high-dimensinal indicators, so as to reduce the dimensions of data [26]. Considering that too many influencing factors weakly related to student grade variables will affect the final prediction effect, it is necessary to use a factor analysis model to process the data characteristics before the experiment. The specific formula of factor analysis is as follows:

$$\begin{array}{c} \left[\begin{array}{c} x_{1}\\ x_{2}\\ \vdots \\ x_{n} \end{array}]=[\begin{array}{cccc} a_{11} & a_{12} & \cdots & a_{1m}\\ a_{21} & a_{22} & \cdots & a_{2m}\\ \vdots & \vdots & \cdots & \vdots \\ a_{n1} & a_{n2} & \cdots & a_{nm} \end{array}][\begin{array}{c} F_{1}\\ F_{2}\\ \vdots \\ F_{n} \end{array}]+[\begin{array}{c} d_{1}\\ d_{2}\\ \vdots \\ d_{n} \end{array}\right] \end{array}$$ (6)

where *x* = (*x*_1_, *x*_2_, ⋯, *x*_*n*_) is an n-dimensional random vector composed of *n* data indicators; *F* = (*F*_1_, *F*_2_, ⋯, *F*_*n*_) is the extracted common factor; *a*_*ij*_ is the factor loading; and *d* is the special factor of *x*. Through the model *x* = *AF* + *d*, the common factor *F* is extracted instead of the original data index *x*, and the final dimensionality reduction among the data is achieved.

#### 3.4.2. GRU model

**Fig 2:**
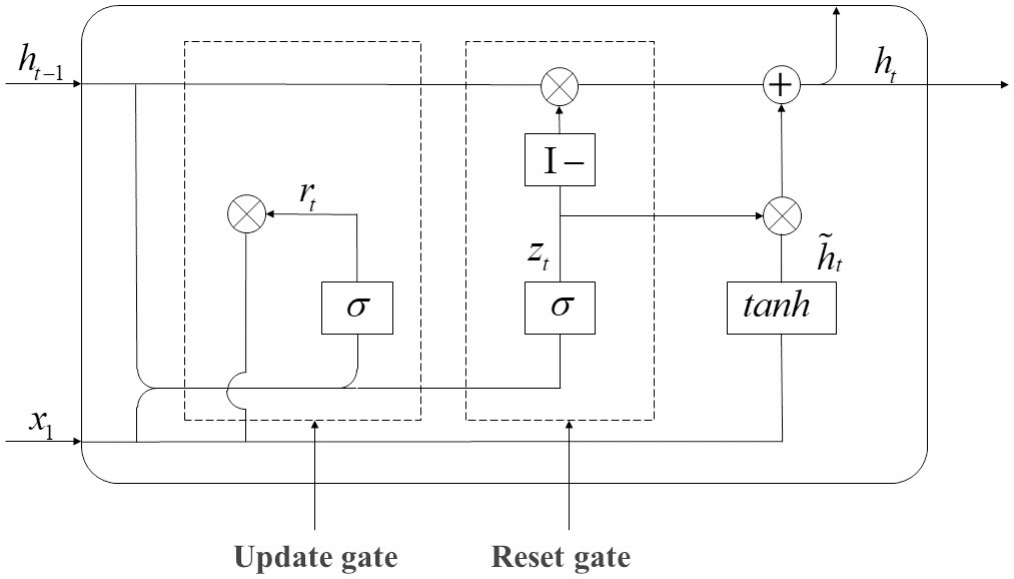
The structure of the GRU model.

GRU neural network model is simpler in structure and has fewer parameters than the LSTM, so the model has a faster training speed and can create a larger network more easily. The update gate can control the degree to which the state information of the previous moment remains in the current state, and the reset gate can control the combination of the current state and the previous information, so the reset gate and the update gate are the core modules of the GRU model [27]. Fig 2 shows the specific structure of the GRU model, and the detailed formula in the model is as follows:

$$\begin{array}{c} r_{t}=\sigma \;(W_{r}\cdot [h_{t-1},x_{t}]) \end{array}$$ (7)

$$\begin{array}{c} z_{t}=\sigma \;(W_{z}\cdot [h_{t-1},x_{t}]) \end{array}$$ (8)

$$\begin{array}{c} {\tilde{h}}_{t}=\text{tanh}\;(W_{\tilde{h}}g[r_{t}\times h_{t-1},x_{t}]) \end{array}$$ (9)

$$\begin{array}{c} h_{t}=(I-z_{t})\times h_{t-1}+z_{t}\times {\tilde{h}}_{t} \end{array}$$ (10)

$$\begin{array}{c} y_{t}=\sigma \;(W_{o}\cdot h_{t}) \end{array}$$ (11)

where *x*_*t*_ is the input vector; *h*(*t* − 1) is the state memory variable at the previous moment; *h*_*t*_ is the state memory variable at the current time; *r*_*t*_ is the update door status; *z*_*t*_ is reset door status; ${\tilde{h}}_{t}$ is the current candidate set status; *y*_*t*_ is the output vector at the current time; *W*_*r*_,*W*_*z*_,$W_{\tilde{h}}$,*W*_*o*_ are respectively the weight parameters of the update gate, reset gate, candidate set, and output vector; I is the unit matrix; *σ* is the *sigmoid* activation function; and *tanh* is the tangent function.

#### 3.4.3. BiGRU model

**Fig 3:**
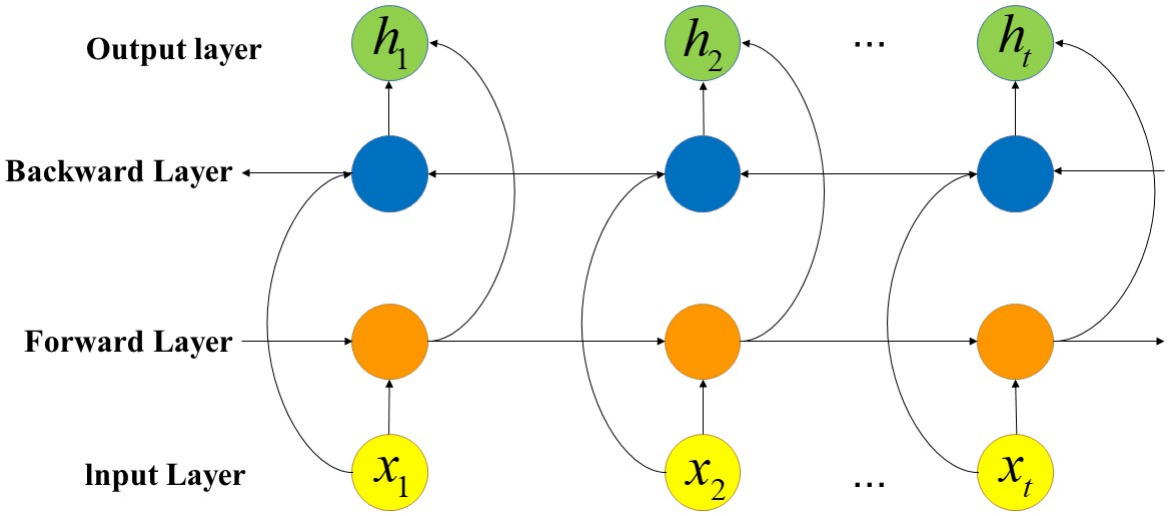
The structure of the BiGRU model.

The BiGRU model is essentially composed of a forward-propagating GRU model and a backward-propagating GRU model [28], whose structure is shown in Fig 3. From it, the BiGRU model can be divided into the input layer, the forward hidden layer, the backward hidden layer, and the output layer, where the forward hidden layer and backward hidden layer are the core of the model. After updating the status of the forward hidden layer and the backward hidden layer, the weighted sum is used to obtain the hidden layer status of BiGRU. The model can improve the recognition efficiency between data, and fully learn the connection between students’ grades before and after. Considering that predicting students’ current grades based on their historical grades will improve the accuracy of the model, this paper uses the BiGRU model to study students’ grade data. The mathematical expressions for the BiGRU model are as follows:

$$\begin{array}{c} {\vec{h}}_{t}=GRU(x_{t},{\vec{h}}_{t-1}) \end{array}$$ (12)

$$\begin{array}{c} {\overset{\leftarrow }{h}}_{t}=GRU(x_{t},{\overset{\leftarrow }{h}}_{t-1}) \end{array}$$ (13)

$$\begin{array}{c} h_{t}=f\;(W_{\vec{h_{t}}}{\vec{h}}_{t}+W_{{\overset{\leftarrow }{h}}_{t}}{\overset{\leftarrow }{h}}_{t}+b_{t}) \end{array}$$ (14)

where ${\vec{h}}_{t}$ and ${\overset{\leftarrow }{h}}_{t}$ are the states of the forward and backward hidden layers at the moment *t*, respectively; $W_{\vec{h_{t}}}$ and $W_{{\overset{\leftarrow }{h}}_{t}}$ are the weights of the forward and backward hidden layer states at the moment *t*, respectively; *b*_*t*_ is the bias of the hidden layer state at moment *t*.

#### 3.4.4. Attention model

**Fig 4:**
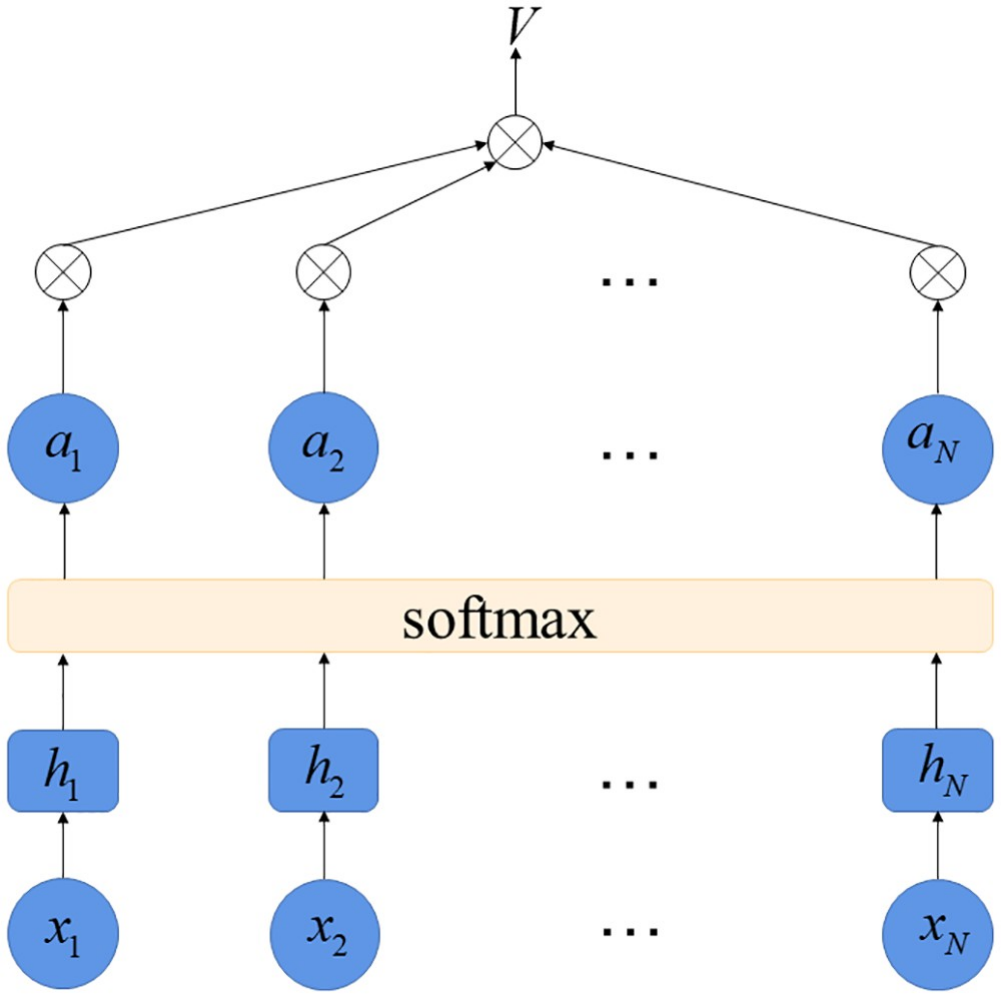
Structural diagram of attention mechanism.

In the practical application of machine learning, the huge model input information may lead to information overload and affect the prediction accuracy of the model. Therefore, the attention mechanism is decided to be used in the neural network for student learning prediction. The attention mechanism allows the model to focus its resources on important information by assigning a series of weighting parameters and reducing the focus on low-relevance information, thus increasing the efficiency of the algorithm [29]. The structure of the attentional mechanism is shown in Fig 4. The calculation of attention value is usually divided into two steps. The first step is to calculate the attention distribution of input information and get the attention score function; The second step is to numerically convert the attention score function through the *softmax* normalization function, and convert the calculated score into a probability distribution with the weighted sum of 1. Therefore, the calculation formulas of the Attention mechanism are as follows:

$$\begin{array}{c} a_{m}=\text{softmax}\;(s(h_{m^{\prime }}h_{t}))=\frac{\text{exp}\;(s(h_{m},h_{t}))}{\sum_{j=1}^{N}\text{exp}\;(s(h_{m},h_{t}))} \end{array}$$ (15)

$$\begin{array}{c} s(h_{m},h_{t})=v^{T}\;\text{tanh}\;(Wh_{m}+Uh_{t}) \end{array}$$ (16)

$$\begin{array}{c} V=\sum_{m=1}^{N}a_{m}h_{m} \end{array}$$ (17)

Where, *a*_*m*_ is the probability that the *m*th input information is obtained; *W*,*U* and *v* are learnable parameters of neural network.

Where *a*_*m*_ is the mth probability of getting input information; *W*,*U* and *v* are learnable parameters of neural network; *N* is the number of data; *x*_1_, *x*_2_, ⋯, *x*_*N*_ are the original data; *h*_1_, *h*_2_, ⋯, *h*_*N*_ are the input information of the neural network; *a*_1_, *a*_2_, ⋯, *a*_*N*_ are the probability that the Nth input information is taken; *V* is the attention value.

#### 3.4.5. Model implementation steps

In the specific implementation of the prediction of students’ learning performance, the operation steps are as follows:

Step 1. After normalization of the original data, KMO and Bartlett’s sphericity tests were performed to determine whether the KMO value was greater than 0.5 and the Bartlett was less than 0.05. If these two conditions are met, it indicates that the student learning data can be characterized for factor analysis [30].

Step 2. Check the cumulative contribution rate of the data, if it is greater than 60%, then you can continue the factor analysis experiment. Indicators of variables with overlapping information and high correlation are grouped into multiple uncorrelated comprehensive factor expressions by means of factor rotation, maximum variance methods, and component score coefficient matrices.

Step 3. Construct the Bidirectional GRU network through the Sklearn package in Python, and add the attention layer to the network to form the BiGRU-attention model for experiments.

Step 4. Collate multiple groups of comprehensive factor expressions and students’ final academic grades into experimental data. Divided the experimental data into a test set and validation set in the ratio of 8 to 2, set appropriate model parameters, and put them into model training to get the final prediction results.

## 4. Results and discussion

When data changes complexly, the single model often cannot achieve ideal prediction results [31]. Considering that the data among students’ grades are irregular jumping, this section focuses on using the combination model to study the score data.

### 4.1. The most import factors impact learners

Considering the ability to process high-dimensional data and sorting characteristic attribute variables according to their importance, this paper chooses the RF model to select the most important factors in the original grade data. By using the RandomForestRegressor package of Sklearn in Python, the weight of each characteristic attribute variable in math courses and Portuguese courses can be calculated respectively. Table 3 contains the top 11 optimal features and their relevancy grade.

**Table 3:** Optimal feature set.

Order	Name	VI(math)	Name	VI(por)
1	G2	0.78134	G2	0.8338
2	Absences	0.13006	Absences	0.02697
3	Age	0.02117	G1	0.0169
4	Health	0.00672	Age	0.01106
5	Mjob	0.00558	Medu	0.00994
6	G1	0.00544	Reason	0.00993
7	Famrel	0.00417	Dalc	0.00991
8	Fedu	0.00356	Freetime	0.00833
9	Walc	0.00329	Famrel	0.00746
10	Studytime	0.00320	Health	0.00555
11	Goout	0.00307	School	0.00516

From Table 3, the five factors that have the highest weight on the final grades have been extracted, namely, grades in two stages, absenteeism, age, health, work and education of the learner’s mother. Among them, past grades and absenteeism times have the strongest correlation with the current grade, so teachers need to consider these two factors more when evaluating students. The development of students is unbalanced at all ages, so teachers should combine the characteristics of students of all ages in the teaching process. In addition, the state of health will directly affect students’ learning efficiency, and the type of work of mothers determines whether they have more time to educate their children. A mother’s educational level indicates whether she can bring more help to her children. Therefore, these five factors have the greatest impact on learners.

### 4.2. Find a model with higher accuracy and wider applicability in the field of learning

#### 4.2.1. RF-BiGRU-attention

**Fig 5:**
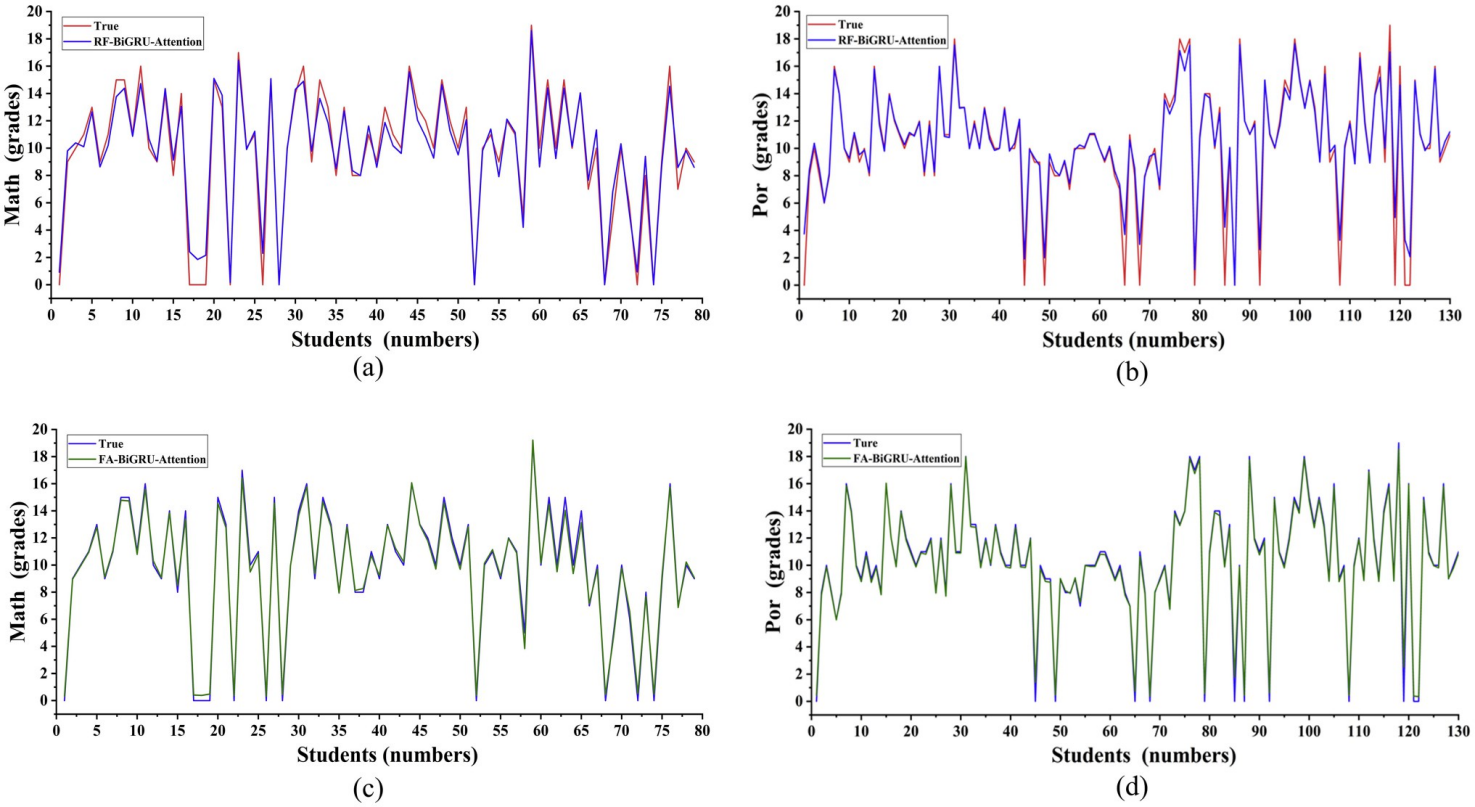
Prediction results of each BiGRU-attention models.

The low correlation variable factors will affect the prediction effect of the experiment [22]. To eliminate the low correlation variable factors, this section used the RF as the feature selection method of the model. Using the 11 feature variables with the highest weights obtained by the RF as the input of the BiGRU-attention model, the prediction results of the student’s grades were obtained after training. From Fig 5a, the fitting between the predicted value of the RF-BiGRU-attention and the real value in students’ Mathematics grades can be observed. Through calculation, the *R*^2^ value of the predicted results for students’ Mathematics grades is 0.966. Similarly, the predicted results of students’ grades in Portuguese under the same experimental approach can be seen in Fig 5b, and the predicted *R*^2^ value is 0.9445. Compared to the prediction results of the mathematical grades, the RF-BiGRU-attention model did not achieve satisfactory predictions in the results of the Portuguese because it did not extract all the important information between the original data. Therefore, the RF-BiGRU-attention model is not universal in the study of students’ learning performance.

#### 4.2.2. FA-BiGRU-attention

To find a general prediction model in the study of students’ learning situations, the FA-BiGRU-attention model was used to continue the study of students’ grades. To facilitate the comparison of the prediction effects of each model in the Sklearn framework, except the random seeds, the experimental parameters for the same courses were set the same, and the parameters were sorted into Table 4. In the study of students’ grades in the mathematics course, the factor analysis model was used as a feature selection method for the model. By inputting 32 independent variable factors into the factor analysis model for the experiment, KMO and Bartlett analysis results with data of 0.614 and 0 were obtained. As the KMO value was greater than 0.5 and the Bartlett value was less than 0.05, it met the conditions for the factor analysis experiment. In further research, the cumulative contribution rate of the data obtained was 63.681%, which was greater than 60%. So, students’ grades in the mathematics course satisfy the prerequisites of the factor analysis model. Through factor rotation, maximum variance, component score coefficient matrix, and other methods, 13 variable indicators were generated finally. Fig 5c shows the training results of the BiGRU-attention model under the input of 13 variable indicators. Through calculation, the R²value of the FA-BiGRU-attention model in predicting the results of students’ mathematics grades was 0.994, which was 2.8% higher than the prediction accuracy of the RF-BiGRU-attention model. As the original dataset of students’ Portuguese language grades could not meet the experimental criteria for the factor analysis model, the 30 variable factors with the largest weights after the random forest calculation were selected as the input to the factor analysis model. Under the same experimental method of factor analysis, ten variable indicators were finally obtained. Fig 5d shows the training results of the FA-BiGRU-attention model for Portuguese language grades under the ten variable indicators. The *R*^2^ value of the model’s prediction result was calculated to be 0.993, which was 4.65% higher than the prediction accuracy of the RF-BiGRU-attention model. Therefore, the FA-BiGRU-attention model can achieve better prediction results than the RF-BiGRU-attention model in the prediction of students’ learning situations.

**Table 4:** Parameters of each model.

RF-BiGRU-attention(math)	RF-BiGRU-attention(por)	FA-BiGRU-attention(math)	FA-BiGRU-attention(por)
Seed = 9	Seed = 9	Seed = 13	Seed = 17
GRU_units = 32	GRU_units = 64	GRU_units = 32	GRU_units = 64
Batch_size = 16	Batch_size = 16	Batch_size = 16	Batch_size = 16
Test_ratio = 0.2	Test_ratio = 0.2	Test_ratio = 0.2	Test_ratio = 0.2
Epoch = 100	Epoch = 120	Epoch = 100	Epoch = 120
Windows = 4	Windows = 4	Windows = 4	Windows = 4

#### 4.2.3. GRU-attention

**Fig 6:**
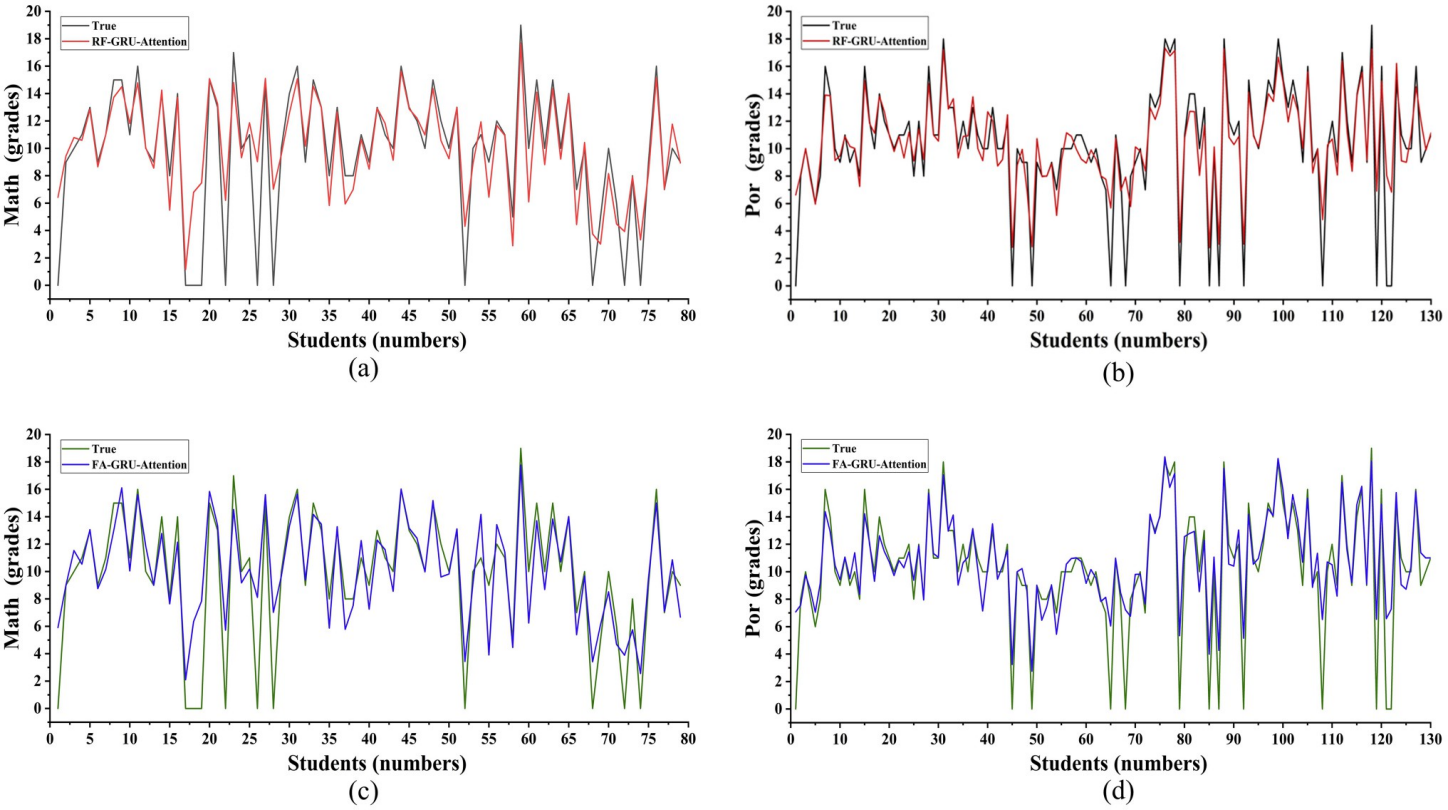
Prediction results of each GRU-attention models.

In the study of students’ grades, the FA-BiGRU-attention model can achieve a better prediction result. To find the best prediction model, the GRU-attention models were used to continue the research under the different feature selection methods. Fig 6a and 6b show the results of the RF-GRU-attention model for predicting students’ grades in Mathematics and Portuguese courses respectively. And Fig 6c and 6d show the results of the FA-GRU-attention model for predicting students’ grades in Mathematics and Portuguese courses respectively. By calculation, the fitting values of the RF-GRU-attention model for predicting students’ grades in Mathematics and Portuguese were 0.745 and 0.804 respectively, while the values of the FA-GRU-attention model were 0.7366 and 0.792 respectively. As the interrelationship between the former and latter students’ grades was not taken into consideration, the GRU-attention model could not achieve the desired prediction results in the study of students’ learning situations.

#### 4.2.4. Experimental comparison and discussion

To verify the effectiveness of the FA-BiGRU-attention model design, this section conducts three adequate experiments to analyze the predictive effect of each model. The first experiment aims to validate the improvement in prediction accuracy brought by the attention mechanism, specifically the prediction effect of the BiGRU model. The second experiment discusses the necessity of the bidirectional gate structure, that is, the predictive effect of the GRU-attention model. The third experiment is designed to demonstrate the predictive effect of the model when both the attention mechanism and the bidirectional gate structure are lacking, i.e. the predictive effect of GRU. To facilitate the comparison of prediction effects, this section also used single models to study students’ learning performance [32]. Table 5 shows the *R*^2^ value of each model’s prediction results under the RF and FA model. Compared with the other model, it can be observed that no matter which data mining method is based on, the BiGRU-attention model has the best fitting effect.

**Table 5:** The fitting results each model.

	RF(math)	RF(por)	FA(math)	FA(por)
BiGRU-attention	0.966	0.9445	0.994	0.993
BiGRU	0.819	0.806	0.841	0.823
GRU-attention	0.745	0.804	0.745	0.792
GRU	0.704	0.706	0.726	0.731
LR	0.7201	0.7901	0.7095	0.7672
RF	0.831	0.7911	0.774	0.7678
LightGBM	0.8105	0.6667	0.827	0.6698
XGBoost	0.772	0.7906	0.8179	0.7228
BP	0.76	0.794	0.7162	0.7592

Tables 6 and 7 respectively show the RMSE and MAE error values of each model under the RF and the FA model. It can be found that the BiGRU-attention model has the smallest prediction error. Therefore, both the attention mechanism and the bidirectional gate structure can significantly improve the prediction accuracy in the prediction of student performance.

**Table 6:** RMSE error values of each model.

	RF(math)	RF(por)	FA(math)	FA(por)
BiGRU-attention	0.8862	1.0371	0.3618	0.3661
BiGRU	1.7985	1.2613	1.9515	1.5532
GRU-attention	2.4296	1.9491	2.4302	2.0071
GRU	2.6167	2.387	2.5183	2.2821
LR	2.5458	2.0177	2.5938	2.125
RF	1.9783	2.0127	2.2875	2.1592
LightGBM	2.0947	2.4962	2.0015	2.4336
XGBoost	2.3796	2.0739	2.0533	2.1746
BP	2.3573	1.9985	2.5634	2.1608

**Table 7:** MAE error values of each model.

	RF(math)	RF(por)	FA(math	FA(por)
BiGRU-attention	0.689	0.5287	0.2972	0.2194
BiGRU	1.1711	0.774	1.2669	0.9294
GRU-attention	1.4928	1.2302	1.6009	1.2027
GRU	1.4765	1.4586	1.6317	1.3046
LR	1.4213	1.1557	1.7013	1.213
RF	1.1846	1.1958	1.357	1.3231
LightGBM	1.4228	1.4323	1.3142	1.4753
XGBoost	1.5165	1.2514	1.2982	1.3444
BP	1.397	1.4546	1.8534	1.23

**Fig 7:**
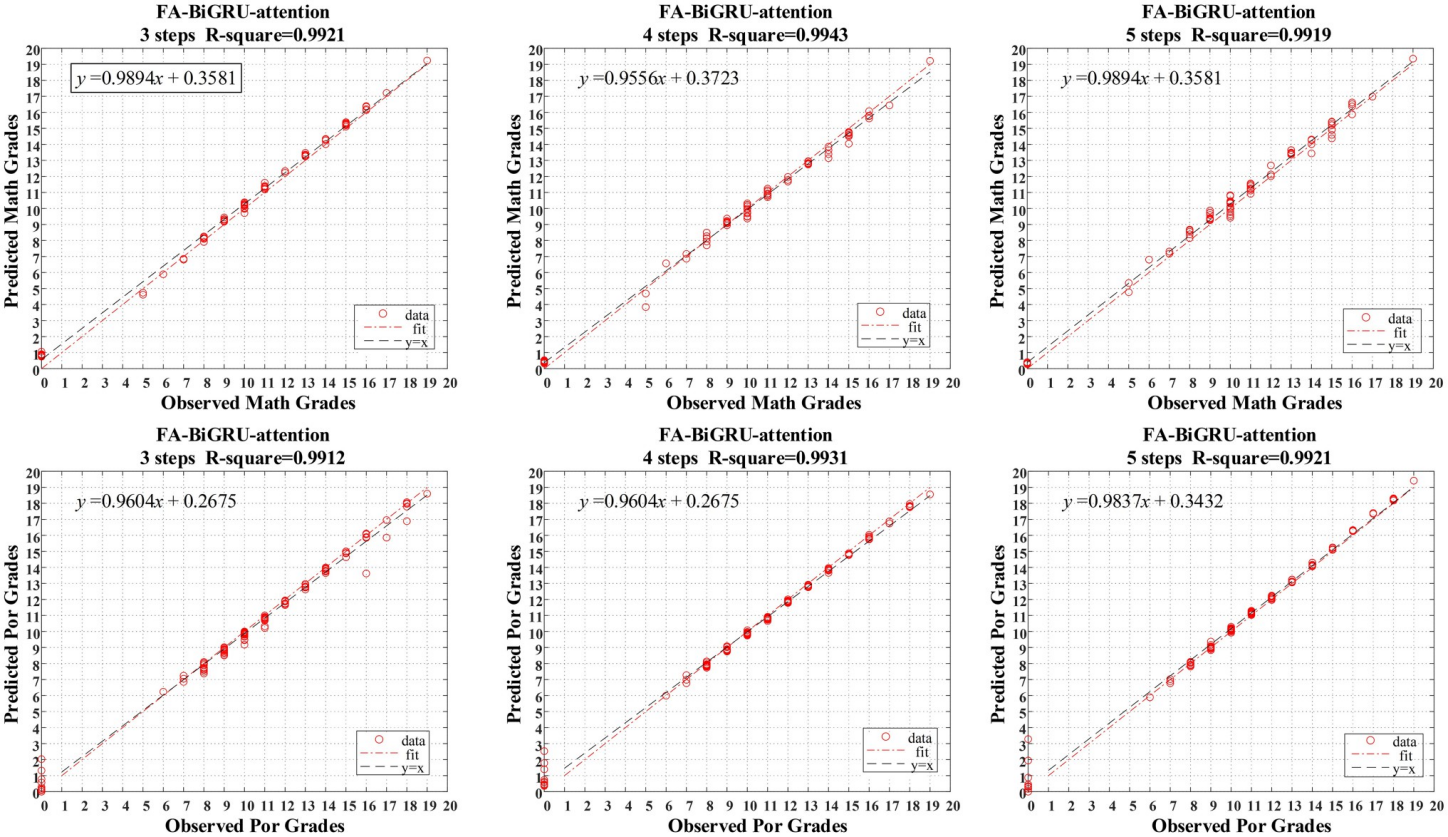
The validation set results under different split function.

Through the above analysis, the BiGRU-attention model achieves the best prediction effect in the study of students’ learning situations. By comparison, the BiGRU-attention based on the FA model is superior to the prediction effect based on the RF model in terms of both fitting effect and error values. To further study the advantages of the FA-BiGRU-attention model, Fig 7 shows the *R*^2^ value of the model’s different multi-step prediction results. From this, it can be observed that the prediction results in each subject can remain relatively stable. By sorting the error values for the different multi-step predictions into Table 8, it is found that the error values predicted by the model are small and remain relatively stable. Therefore, the FA-BiGRU-attention model has a high practicability in the prediction of students’ learning situations and has the potential to be applied to other research fields.

**Table 8:** The fitting results under different split function.

	FA-BiGRU-attention	3 steps	4 steps	5 steps
math	RMSE	0.4088	0.3618	0.4324
	MAE	0.3364	0.2972	0.3813
por	RMSE	0.4134	0.3661	0.3913
	MAE	0.242	0.2194	0.2019

### 4.3. The needs of learners

**Fig 8:**
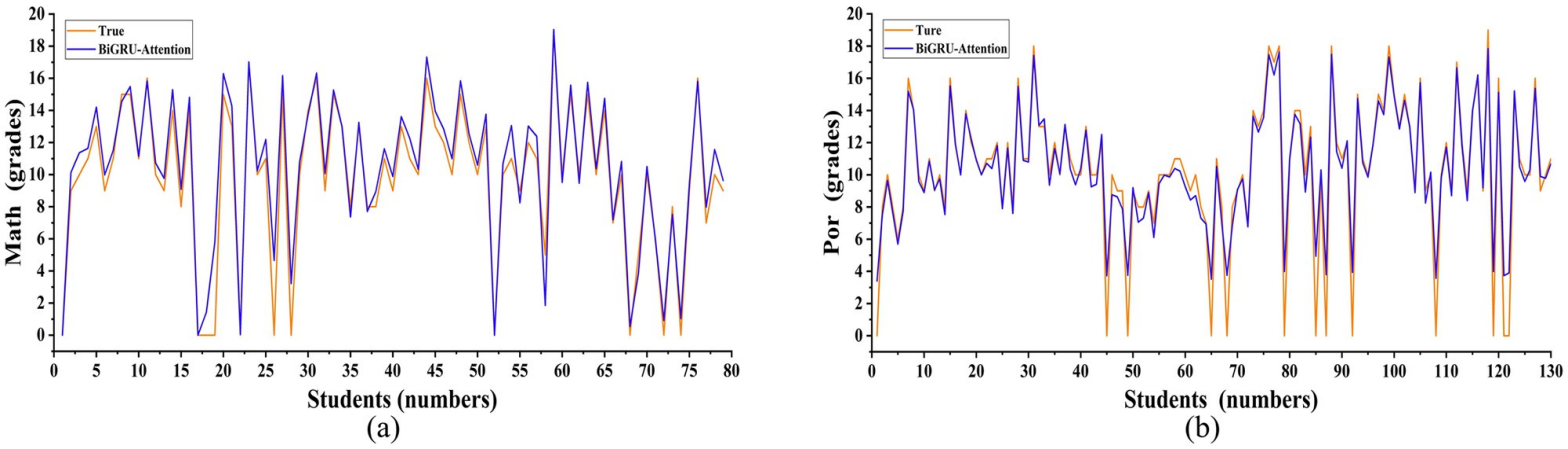
Prediction results of the BiGRU-attention on the raw data.

Through the analysis and discussion of the above experimental results, the combination model can achieve a better prediction effect. To find out what learners need most, the BiGRU-attention model was used to research the raw data on students’ grades. By setting the same model parameters in the previous experiment, the predicted results for students’ grades in mathematics and Portuguese can be seen in Fig 8. By calculation, the *R*^2^ value of the BiGRU-attention model for predicting students’ grades in Mathematics and Portuguese were 0.907 and 0.914 respectively. Since the prediction effect of the BiGRU-attention model on the original data is lower than the model after feature selection, it can conclude that data mining on multivariate data can improve the prediction effect of the model.

Therefore, all of us can know that what learners need most is personalized feedback information after data mining. Educational data mining can select characteristics of students’ contextual characteristics and historical performance to obtain more beneficial information for the student’s development and to help predict the student’s academic performance. By adjusting their learning strategies and improving their work efficiency, personalized feedback information can help machine learning achieve better prediction effects and help learners progress toward their goals as much as possible.

## 5. Conclusion

Through data mining and machine learning technology, this paper proposes a performance prediction model based on background features and learning performance. By selecting the attribute features with a high correlation to the prediction target, such as 11 attributes extracted by RF and 13 attributes extracted by FA, the prediction accuracy is improved. Therefore, the accuracy of prediction results depends not only on the performance of the predictive model, but also on the number and weight of attributes.

The results of the above experiments fully answer the three research questions posed in the introduction. Five factors with the highest weight were extracted from the analyzed data, which were the results of the two stages, absenteeism rate, age, health status, mother’s work and education level. Past grades and absences are a direct reflection of previous periods of performance and therefore have the strongest correlation with current grades. Therefore, teachers need to take these two factors more seriously in their assessment of students. Students’ development is uneven at all ages. In the teaching process, teachers should teach with the characteristics of students at this age, take their current level as a starting point, grasp the “nearest development zone” and explore the potential level of students. In addition, students’ health has a direct impact on their state of learning and thus on their effectiveness. The type of work a mother does represents whether they have more time to manage and educate their children, and the educational level of a mother represents whether it can bring a better impact and help their children more. What do they expect technology to bring to students? The use of technology can effectively predict students’ future performance and formulate the best educational strategy for each student by making appropriate interventions and adjustments based on relevant factors.

By adding FA into the BiGRU-attention model in an innovative way, the contribution of this research lies in developing a new prediction model of academic performance, achieving good prediction results, and answering the questions raised in the introduction. This can help teachers and students find the root causes of problems, thus solving problems more pertinently, and providing decision support for students’ personalized education strategies.

This study also has some limitations. It is meaningful to use data mining technologies to predict students’ performance, but it also brings some risks. First, data mining needs more quantity and more diverse data types, this study only extracts 32 representative attributes according to students’ learning situations, and does not analyze other features. Secondly, this paper only selects nine representative algorithms for analysis and research. With the rapid development of machine learning, there will be models with higher accuracy, better stability and wider applicability in the future remain to be studied.
